# Supplementary material for: Causal Relationship between Aging and Anorexia Nervosa: A White-Matter-Microstructure-Mediated Mendelian Randomization Analysis
Source: Biomedicines. 2024 Aug 16;12(8):1874. doi: 10.3390/biomedicines12081874 (PMC11351342; doi:10.3390/biomedicines12081874)
Supplement: Supplementary file 1 [file biomedicines-12-01874-s001.zip › Supplementary Figure/Figure S4/Singe SNP plot of MR analysis for TL to ubm-b-1825.pdf]

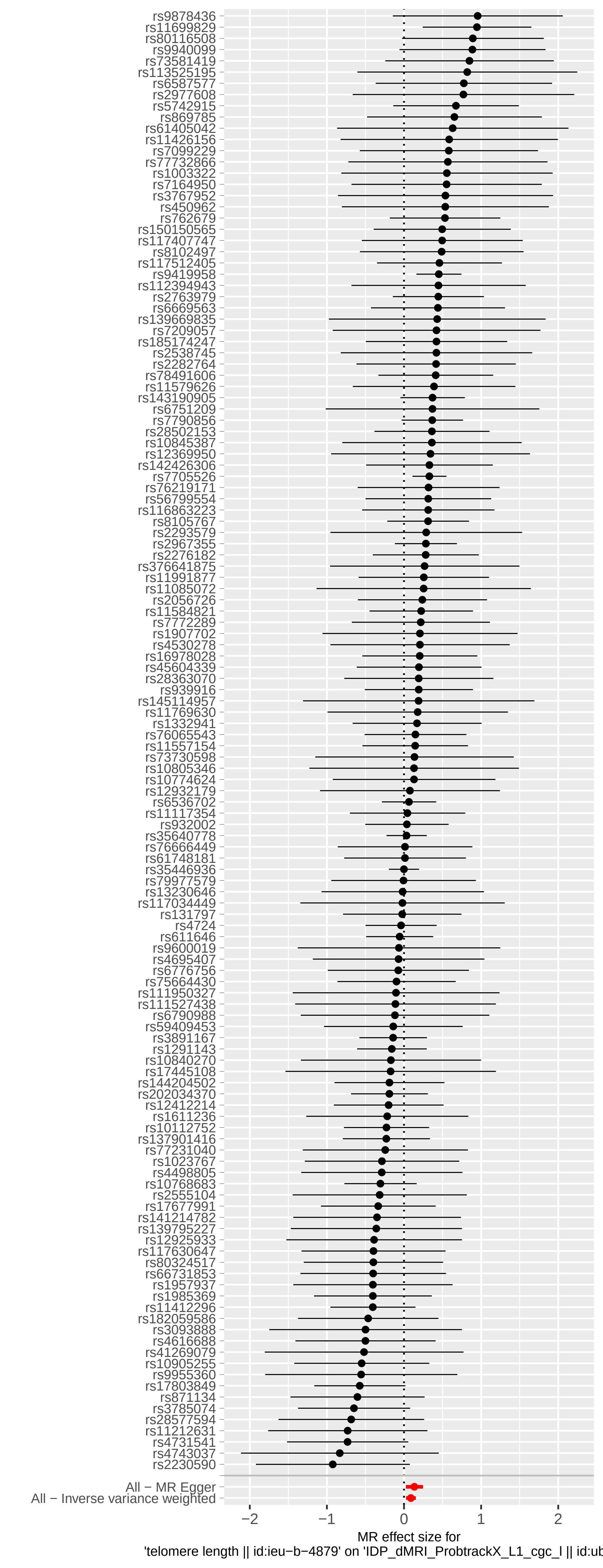

rs9878436
rs11699829
rs80116508
rs9940099
rs73581419
rs113525195
rs6587577
rs2977608
rs5742915
rs869785
rs61405042
rs11426156
rs7099229
rs77732866
rs1003322
rs7164950
rs3767952
rs450962
rs762679
rs150150565
rs117407747
rs8102497
rs117512405
rs9419958
rs112394943
rs2763979
rs6669563
rs139669835
rs7209057
rs185174247
rs2538745
rs2282764
rs78491606
rs11579626
rs143190905
rs6751209
rs7790856
rs28502153
rs10845387
rs12369950
rs142426306
rs7705526
rs76219171
rs56799554
rs116863223
rs8105767
rs2293579
rs2967355
rs2276182
rs376641875
rs11991877
rs11085072
rs2056726
rs11584821
rs7772289
rs1907702
rs4530278
rs16978028
rs45604339
rs28363070
rs939916
rs145114957
rs11769630
rs1332941
rs76065543
rs11557154
rs73730598
rs10805346
rs10774624
rs12932179
rs6536702
rs11117354
rs932002
rs35640778
rs76666449
rs61748181
rs35446936
rs79977579
rs13230646
rs117034449
rs131797
rs4724
rs611646
rs9600019
rs4695407
rs6776756
rs75664430
rs111950327
rs111527438
rs6790988
rs59409453
rs3891167
rs1291143
rs10840270
rs17445108
rs144204502
rs202034370
rs12412214
rs1611236
rs10112752
rs137901416
rs77231040
rs1023767
rs4498805
rs10768683
rs2555104
rs17677991
rs141214782
rs139795227
rs12925933
rs117630647
rs80324517
rs66731853
rs1957937
rs1985369
rs11412296
rs182059586
rs3093888
rs4616688
rs41269079
rs10905255
rs9955360
rs17803849
rs871134
rs3785074
rs28577594
rs11212631
rs4731541
rs4743037
rs2230590
All − MR Egger
All − Inverse variance weighted
−2
−1
0
1
2
MR effect size for
'telomere length || id:ieu−b−4879' on 'IDP_dMRI_ProbtrackX_L1_cgc_l || id:ub
